# Pilot Implementation of the NeuroSense PremmieEd Parenting Educational Programme for Parents with Infants in the Neonatal Intensive Care Unit: A Sequential Cohort Design

**DOI:** 10.3390/children12121636

**Published:** 2025-12-01

**Authors:** Welma Lubbe, Kirsten A. Donald

**Affiliations:** 1 Division of Developmental Paediatrics, Department of Paediatrics & Child Health, Red Cross War Memorial Children’s Hospital, Neuroscience Institute, University of Cape Town, Cape Town 7700, South Africa; kirsty.donald@uct.ac.za; 2 NuMIQ Research Focus Area, Faculty of Health Sciences, North-West University, Potchefstroom 2531, South Africa

**Keywords:** neurodevelopmental care, preterm infant, parent education intervention, programmes, parental knowledge, KPIB (Knowledge of Preterm Infant Behaviour), PSS, NICU (Parental Stressor Scale, Neonatal intensive Care Unit), maternal stress

## Abstract

**Highlights:**

**What are the main findings?**

- Structured parenting education, particularly when combined with facilitated sessions, showed a trend towards modest improvements in maternal knowledge of preterm infant behaviour in the NICU, although changes were not statistically significant.
- Parental stress increased in all groups during the NICU stay, with mothers in the facilitated intervention group experiencing the smallest increase.

**Implication of the main findings:**

- Facilitated educational interventions may support early parenting and enhance caregiver understanding during the NICU stay.
- Such interventions could help buffer parental stress and guide the development of psychosocial support strategies in NICUs.

**Abstract:**

**Introduction:** Preterm birth and neonatal intensive care unit (NICU) admission may elevate parental stress and impair early parent–infant interaction. In low-resource settings, where staff and resources are limited, parental education programmes are often also limited, further complicating care engagement. This study piloted the NeuroSense PremmieEd parenting educational programme to assess its preliminary impact on maternal stress and knowledge in a South African public-sector NICU. **Objectives:** This study aimed to pilot a contextually relevant parenting education intervention to enhance parental understanding of preterm infant behaviour, strengthen parents’ capacity to interpret and respond sensitively to infant cues, and reduce parental stress during NICU admission. **Methodology:** This sequential cohort pilot study involved 60 mothers of preterm infants (gestational age, 24–36 weeks) admitted to two comparable NICUs. Mothers (aged 18–45 years) were allocated to three groups. Arm 1 received standard care (*n* = 20), Arm 2 received a printed educational booklet (*n* = 20), and Arm 3 received the booklet plus a facilitated education session (*n* = 20). Pre- and post-intervention data were collected using the Knowledge of Preterm Infant Behaviour (KPIB) questionnaire and the Parental Stressor Scale: NICU (PSS:NICU). Descriptive statistics were used to explore differences between arms. **Results:** Knowledge scores measured with the KPIB tool showed a positive trend in all groups, with the greatest improvement observed in Arm 3; however, these changes were not statistically significant (*p* = 0.176). Maternal stress measured using the PSS:NICU increased significantly over time across all groups (F(1, 57) = 8.40, *p* = 0.005), with Arm 3 consistently reporting the lowest stress at both timepoints. **Discussion:** The facilitated intervention was associated with a trend towards improved maternal knowledge of preterm infant behaviour. This pilot study highlighted the potential of structured and culturally relevant education to support early parenting in a public-sector NICU in South Africa. Maternal stress levels remained high across all groups. While this finding may be due to parents’ experience of changes in infant medical condition, fatigue, and other factors, these were not investigated in this study and therefore warrant further exploration in future work. **Conclusions:** The NeuroSense PremmieEd programme shows promise in improving maternal understanding of preterm infant behaviour. The results highlight the need for further adaptation of content delivery, inclusion of diverse populations (e.g., by preterm category) and scalable, low-resource approaches to improve engagement and long-term outcomes.

## 1. Background

The neonatal intensive care unit (NICU) plays a critical role in supporting the survival of preterm infants through advanced medical interventions and technologies designed to support physiological processes [1]. However, despite its lifesaving benefits, the NICU environment can be highly stressful for both infants and their parents [2]. Parents often experience elevated anxiety and emotional distress due to the uncertainty of their infant’s condition, compounded by the unfamiliar and clinical nature of the NICU setting [1,3].

Preterm birth interrupts the natural developmental process, removing the infant from the protective intrauterine environment and the physiological regulation typically provided by the mother [4]. This early separation can disrupt the development of the parent–infant bond and interfere with co-regulation mechanisms vital for emotional and neurological development [5,6].

Parental education is an important potential intervention strategy to mitigate these challenges [7]. Increasing parents’ understanding of preterm infant behaviour helps them interpret their infants’ cues, respond more effectively, and reduce feelings of helplessness and anxiety [1,8,9]. Knowledge about developmental needs, stress signals, and caregiving techniques, such as appropriate positioning, comfort strategies, and recognition of signs of overstimulation, can foster emotional resilience, counter feelings of isolation, support realistic expectations, and empower parents to respond in a nurturing and active manner [1,4,10,11,12,13].

Parental education not only benefits parents but may also have a positive impact on their infants [14,15,16,17]. Informed and engaged parents are more likely to adopt and sustain positive caregiving behaviours, including kangaroo care and responsive interactions. These practices are crucial for the healthy emotional and neurological development of the infant [18,19,20]. They also enhance bonding [15,21], improve sleep regulation and quality [22,23] and support overall brain development in preterm infants [18,23]. With knowledge of infant behaviour and development, parents are better equipped to implement care techniques that support co-regulation, contributing to their infant’s physical and neurological growth [24].

Evidence-based parenting educational programmes grounded in family-centred care (FCC) have demonstrated positive outcomes for both infants and parents [25]. Notable examples include the Creating Opportunities for Parent Empowerment (COPE) programme [26] and the Alberta Family Integrated Care (FICare) psychoeducational intervention [27]. Building on this foundation, the NeuroSense PremmieEd Parenting Educational Programme was developed in South Africa. This programme draws on global literature and qualitative data from mothers of preterm infants within a selected context of the South African public healthcare sector, to support contextual relevance.

The study was conducted at two public-sector referral hospitals located in the Dr Kenneth Kaunda District of South Africa’s North West province, a semi-urban region where residents often move between rural and urban areas to access healthcare services, making use of public and private providers. The selected hospitals provide similar services with hospital A’s neonatal unit comprising 8 NICU beds, 9 high-care beds and 28 low-care beds, admitting an average of 66 neonates per month [28]. Hospital B is a regional hospital with 6 NICU beds, 10 high-care and 4 low-care (kangaroo-mother-care) beds. Both units provide care for very-low-birthweight preterm infants (from 24 weeks’ gestation), late preterm infants, and high-risk full-term neonates.

This pilot study was conducted to inform the refinement of a larger educational programme. It reports on the pilot implementation of the NeuroSense PremmieEd Parenting Educational Programme in tertiary-level NICUs in the North West province of South Africa. While the broader study explored the feasibility and acceptability of the programme, this paper focuses on the preliminary effects of the intervention on parental knowledge and stress, using a sequential cohort design.

### Study Aim

This study aimed to pilot a contextually relevant parenting educational programme to enhance parental understanding of preterm infant behaviour, strengthen parents’ capacity to interpret and respond sensitively to infant cues, and reduce parental stress during NICU admission.

## 2. Methods

### 2.1. Design

Informed by the frameworks of Kleberg et al. [29] and Browne and Talmi [30], our study employed a sequential cohort design comprising three arms: standard care, booklet-only, and full intervention. Standard care in our setting did not include parenting education interventions.

### 2.2. Recruitment of Participants

Following ethical approval, a research assistant approached mothers with NICU babies that were stable and older than 24 h. The assistant explained the study, provided an information brochure and a copy of the informed consent document, and addressed any questions that arose. Mothers who agreed to participate signed an informed consent form and completed biographical and other assessment questionnaires as soon as possible after enrolment.

#### 2.2.1. Inclusion Criteria

Mothers were eligible if the infant(s) were inborn, singleton or multiple, born before 37 weeks gestation (≤36 weeks, 6 days) and expected to remain in the NICU for at least seven days. Mothers were enrolled at least 24 h postnatally. No fathers were actively involved in these units and were therefore not involved in this pilot. In the case of infant death, mothers could choose to remain enrolled or withdraw from the study. Eligible mothers were 18 years or older, provided written informed consent, and were proficient in at least one of the local languages, namely, English, Setswana, or Afrikaans.

#### 2.2.2. Exclusion Criteria

Teen mothers under 18 years of age were excluded due to their unique parenting needs. Infants with major malformations or conditions requiring surgery were also excluded. Additionally, parents with a history of drug addiction, psychosis, or severe mental illness (self-reported) were not eligible to participate.

#### 2.2.3. Sample Size

The sample goal of 30 participants (10 in each group) per hospital was predetermined based on previous comparable studies [29,30]. Kleberg et al. [29] in their study enrolled 10 mothers each in the control and experimental groups, comparing mothers’ attitudes and apprehension of their maternal role, perception of their infant, and neonatal care when receiving the Newborn Individualized Developmental Care and Assessment Program (NIDCAP) intervention versus no intervention (a pilot study). Browne and Talmi [30], on the other hand, included 84 high-risk mother-infant dyads which were randomly assigned to two intervention and one control group (full randomised controlled trial). In their study, they examined how family-based interventions in the NICU changed parental knowledge and behaviours and decreased stress. We recruited 20 participants (10 per hospital) in each of the three arms (*n* = 60) to determine feasibility.

### 2.3. Procedures and Data Collection

#### Measures

Demographics and clinical descriptive data

Biographical data were collected from the mothers through self-administered questionnaires. Parental variables included marital status, parity, gravidity, age, ethnicity, pregnancy and birth history, type of delivery, and educational level (see Table 1: Biographical). Infant characteristics included gestational age, birth weight and size, head circumference at birth, APGAR, and gender [29].

The Knowledge of Preterm Infant Behaviour Scale (KPIB) [30] uses 36 multiple-choice questions to elicit parents’ knowledge of their preterm infant in two domains of infant behaviour: (1) knowledge of infant reflexes, physical responses to stimuli, motor activity, sleep–wake states and social interaction, and (2) parental knowledge regarding optimal interaction times and how to help develop self-regulatory mechanisms in the infant [30]. 

**Figure 1:**
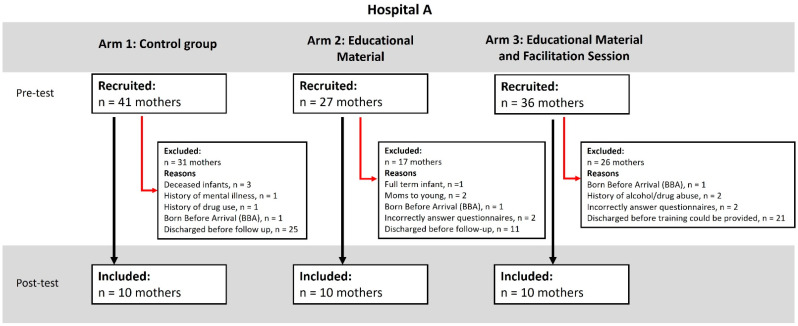
CONSORT diagram of recruitment for Hospital A.

**Figure 2:**
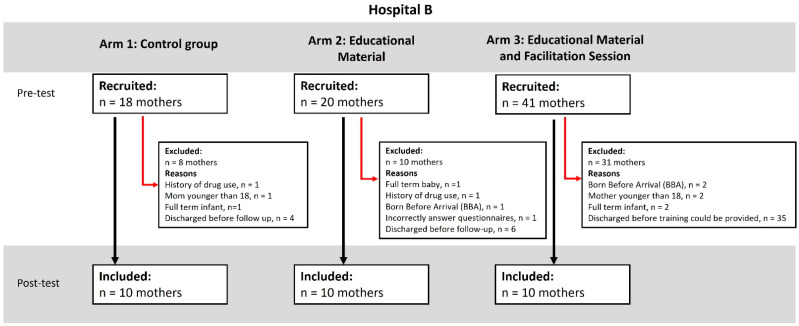
CONSORT diagram of recruitment for Hospital B.

The Parental Stressor Scale: Neonatal Intensive Care Unit (PSS:NICU) [31] is a self-report questionnaire, which measures parental stress following preterm birth and the subsequent admission of the neonate to the NICU. Parents indicated how stressful they experienced situations using a five-point Likert scale with ratings of 1 = not at all stressful (the experience did not cause you to feel upset, tense, or anxious), 2 = a little stressful, 3 = moderately stressful, 4 = very stressful, and 5 = extremely stressful. ‘Not applicable’ could be selected for items that parents had not experienced. The PSS:NICU includes 34 items and consists of three subscales: ‘Sights and Sound’ consisting of 5 items, ‘Parental Role Alteration’ with 14 items and ‘Looks and Behave’ of the child with 15 items [30]. The scale demonstrated a high test–retest reliability. Excellent psychometric properties were shown by Ionio and colleagues [32,33]. The recruitment in this study is presented using CONSORT diagrams (see Figures 1 and 2: CONSORT diagrams of recruitment).

Both the KPIB and PSS:NICU instruments were available in English only. For mothers who preferred to complete the questionnaires in Setswana or Afrikaans, bilingual trained staff provided verbal translation. At this stage, formal translation and validation of the tools were not feasible due to cost and resource constraints. However, feedback from this pilot study may inform the refinement of the tools and the development of translated and culturally adapted versions for future large-scale or randomised controlled trials.

### 2.4. Arm Assignment

A total of 60 mothers participated in the study. Participants were sequentially recruited into three study arms, beginning with Arm 1 (*n* = 20), followed by Arm 2 (*n* = 20), and finally, Arm 3 (*n* = 20), with 10 mothers per arm per hospital. Each arm was completed before the next was enrolled. All participants completed a biographical questionnaire along with the KPIB and PSS:NICU tools at baseline (pre-test) and again, one week later (post-test).

#### 2.4.1. Arm 1: Control (Standard Care)

The control arm (*n* = 20) received the unit’s standard care, which typically involved incidental education or bedside guidance from staff, depending on availability. Mothers visited during scheduled feeding times and participated in basic caregiving tasks such as nappy changes, breastmilk expression, or breastfeeding, if appropriate, but did not necessarily remain with their infants between feedings. Although the physical environment remained unchanged, some elements of neurodevelopmental support (e.g., comfort during handling and kangaroo care) may have occurred spontaneously but were not promoted or restricted by the study protocol.

#### 2.4.2. Arm 2: Educational Booklet

Following the completion of the control arm, the second arm (*n* = 20) was enrolled. In addition to standard care, mothers received an educational booklet immediately after enrolment in English or Setswana (based on their preference, with no mothers requesting materials in Afrikaans), which they were encouraged to read. They also completed the biographical questionnaire and the initial measures of KPIB and PSS:NICU. The mothers had the booklet with them for at least one week before the follow-up measures were taken, as explained during the informed consent process.

#### 2.4.3. Arm 3: Booklet and In-Person Session

The final arm (*n* = 20) received both the educational booklet (following the pre-test measures) and a 60 min in-person training session, led by a healthcare professional with neurodevelopmental expertise. Sessions were delivered in small groups of 2 to 4 mothers within one week of enrolment. The research team trained healthcare professionals to present the standardised printed booklet and guide discussions using a structured set of questions. Presenters included a registered nurse with NICU experience in one hospital and social workers in the other (on request of the hospital).

### 2.5. Data Analysis

Data were initially collected using hard copy questionnaires and captured on an Excel spreadsheet for descriptive statistical analysis. Each participant was assigned a unique identifier to facilitate matched comparisons between pre-test and post-test responses.

Descriptive statistics were used to summarise the sample’s demographic characteristics (reported as frequencies and percentages for categorical variables) and to describe knowledge and stress scores at pre-test and post-test (reported as means, standard deviations, and percentages where applicable). Changes in scores were examined both within each study arm over time and between the three arms. A mixed-design ANOVA (SPSS version 30) was conducted with time (Pre, Post-) as the within-subject factor and group (arms 1, 2, and 3) as the between-subject factor.

### 2.6. Ethical Considerations

The study received ethical approval from the Health Research Ethics Committee of the University of Cape Town (UCT-647/2021), followed by approvals from the North West Province provincial and hospital managements.

## 3. Results

Biographical data covering the characteristics of the participant and infant were self-reported and are summarised in Table 1. In total, 183 participants were recruited, of whom 123 were excluded or lost to follow-up due to reasons such as infant death, changing infant condition, or discharge before completion of the intervention. The reasons for exclusion are shown in Figure 1 and Figure 2. A total of 60 mothers participated in the study, 30 from Hospital A and 30 from Hospital B, with 10 in each arm (see Figure 1 and Figure 2).

### 3.1. Maternal Characteristics

#### 3.1.1. By Hospital

Maternal characteristics by hospital are presented in Table 1. Across both hospitals, most mothers were single (Hospital A: 96.7%; Hospital B: 80.0%). A small proportion of mothers were married (Hospital A: 3.3%; Hospital B: 16.7%) or separated (Hospital B only: 3.3%). The majority of participants identified as African (Hospital A: 100%; Hospital B: 93.3%), with two mothers in Hospital B identifying as Coloured (*n* = 1) or White (*n* = 1).

The mean maternal age was similar between hospitals, with Hospital A at 30.3 years (SD = 7.0; range: 18–45) and Hospital B at 29.7 years (SD = 7.0; range: 20–43). Mothers reported an average of 3.0 pregnancies in Hospital A (SD = 1.7; range: 1–8) and 2.4 in Hospital B (SD = 1.5; range: 1–9). Secondary education was most common (Hospital A: 73.3%; Hospital B: 76.7%), with small numbers completing certificates or diplomas, and very few holding a degree (Hospital A: 10.0%; Hospital B: 3.3%).

Pregnancy and birth complications were reported in both hospitals. In Hospital A, 36.7% of mothers reported complications, most frequently high blood pressure (16.7%) and foetal distress (10.0%). In Hospital B, 56.7% reported complications, including high blood pressure (23.3%) and foetal distress (13.3%). Vaginal delivery was more common in Hospital A (50.0%), whereas emergency caesarean section was more prevalent in Hospital B (56.7%).

#### 3.1.2. By Study Arm

Maternal characteristics by study arm are summarised in Table 2. Age was relatively consistent across arms, ranging from a mean of 26.4 to 31.5 years (SD range: 5.7–7.8). The majority of mothers in all arms were single (Arm 1: 85.0%; Arm 2: 85.0%; Arm 3: 95.0%). Educational attainment was also similar across arms, with secondary education the most common (Arm 1: 75.0%; Arm 2: 70.0%; Arm 3: 80.0%). Mean number of pregnancies ranged from 2.4 to 3.2 across arms (SD range: 0.9–2.4). The distribution of pregnancy complications and mode of delivery was comparable across arms, with no notable imbalances.

### 3.2. Infant Characteristics

#### 3.2.1. By Hospital

Infant characteristics by hospital are shown in Table 3. Mean gestational age was slightly lower in Hospital A (30.1 weeks, SD = 2.7; range: 24–35) compared with Hospital B (31.1 weeks, SD = 2.6; range: 26–36). Hospital A had a slightly higher birthweight, averaging 1402 g (SD = 380; range: 705–2500 g), compared to Hospital B averaging 1300 g (SD = 428; range: 730–2390 g). Head circumference data were more complete in Hospital A (*n* = 26; mean = 27.9 cm, SD = 3.0; range: 23–37) than in Hospital B (*n* = 7; mean = 28.6 cm, SD = 3.3; range: 23–34).

Apgar scores at 1 min averaged 6.9 (SD = 2.1) in Hospital A (*n* = 25) and 7.4 (SD = 2.6) in Hospital B (*n* = 31). At 5 min, scores averaged 8.5 (SD = 1.1, *n* = 24) in Hospital A and 8.3 (SD = 2.3, *n* = 31) in Hospital B. Sex distribution was balanced across the sites, with 41.2% male in Hospital A and 47.1% male in Hospital B.

#### 3.2.2. By Study Arm

Infant characteristics by study arm are summarised in Table 4. The mean gestational age ranged from 29.8 weeks (SD = 2.6) in Arm 1 to 31.3 weeks (SD = 2.4) in Arm 2. Birth weight ranged from a mean of 1249 g (SD = 269) in Arm 1 to 1424 g (SD = 401) in Arm 2, with Arm 3 at 1361 g (SD = 498). Head circumference means ranged between 27.4 cm and 30.1 cm across arms; however, available data varied (Arm 1: *n* = 18; Arm 2: *n* = 13; Arm 3: *n* = 12). Apgar scores at 1 min ranged from 7.0 to 7.3 across arms (SD range: 2.1–2.5), and 5 min scores ranged from 8.3 to 8.4 (SD range: 1.7–2.3). Sex was balanced across arms, with male representation between 36.4% and 47.8%.

Overall, infant baseline characteristics showed expected variability for a preterm NICU population and were broadly comparable across hospitals and arms.

### 3.3. Parental Knowledge (KPIB)

Descriptive statistics for parental knowledge scores are presented in Table 5. Baseline (pre-test) KPIB scores were comparable across the three study arms, with mean scores ranging from 30.6% to 33.6%. Following the intervention, small changes in knowledge were observed. Arm 3 demonstrated the greatest increase from pre- to post-test (+4.0%), followed by Arm 2 (+1.9%), whereas Arm 1 showed a slight decline (–0.7%). When pooled across sites, overall knowledge increased modestly by +1.7%.

A mixed-design ANOVA showed that the change in KPIB scores over time was not statistically significant, F(1, 57) = 1.88, *p* = 0.176, ηp^2^ = 0.03. There were no significant differences between study arms, F(2, 57) = 0.19, *p* = 0.828, ηp^2^ = 0.01, and no significant Time × Group interaction, F(2, 57) = 0.78, *p* = 0.462, ηp^2^ = 0.03 (Table 6). These findings indicate that the intervention did not produce differential effects on parental knowledge, and overall improvements were minimal across all arms.

### 3.4. Parental Stress (NICU:PSS)

Parental stress levels increased from pre- to post-test across all study arms (Table 7). At baseline, stress scores ranged from 52% to 76% across the sites and study arms. Arm 2 showed the largest increase in stress from pre- to post-test (+10.9%), followed by Arm 1 (+4.9%) and Arm 3 (+3.7%). Overall, parental stress increased by +6.3% across the full sample.

The mixed-design ANOVA identified a significant main effect of Time, indicating that parental stress increased over the study period, F(1, 57) = 8.40, *p* = 0.005, ηp^2^ = 0.13. However, there were no significant Group differences in stress, F(2, 57) = 0.75, *p* = 0.479, ηp^2^ = 0.03, and no significant Time × Group interaction, F(2, 57) = 0.99, *p* = 0.378, ηp^2^ = 0.03 (Table 8). These results suggest that stress increased irrespective of intervention arm, and the magnitude of change did not differ across groups.

## 4. Discussion

This pilot study demonstrated the feasibility of implementing a contextually relevant parenting education programme in public-sector NICUs in South Africa. A sequential cohort design was used in two comparable hospitals, each following the same three-arm structure. This approach minimised the risk of contamination between arms, which was critical given that all infants within each site were admitted to shared neonatal units [34].

Participants in this study reported elevated maternal stress, which likely shaped both their experiences and engagement with the intervention materials. The majority of participants were single mothers (*n* = 53), which may have contributed to higher stress due to reduced partner support. Although socioeconomic status was not directly assessed, recruitment from public-sector hospitals likely reflects a predominance of lower-income families.

Parental stress, as measured by the PSS:NICU, increased across the sample from pre- to post-test, regardless of whether the parents received standard care or educational intervention. Subscale analyses indicated that most increases were driven by perceptions of environmental stressors and infant condition, while stress related to the parental role remained relatively stable across arms. One possible explanation for this pattern is that the emotional and physical demands of a high-intensity NICU setting could limit parents’ readiness to engage with educational content, as suggested by prior research [35,36,37].

These insights directly inform the refinement of the forthcoming larger programme. Interventions may need to be flexible and minimally burdensome, with shorter or modular sessions that align with periods when parents are most able to participate. Alternative delivery methods, such as brief bedside teaching, digital resources, or take-home materials, could increase accessibility. Incorporating supportive measures, such as emotional check-ins or peer support, alongside educational content, may also help parents engage more effectively despite high stress.

Parental knowledge gains appeared greatest in Arm 3, where parents received both the educational booklet and a facilitated session, although these differences were not statistically significant. This trend aligns with previous research suggesting that facilitated interactive learning formats are more effective than passive information delivery in helping parents understand and interpret preterm infant behaviour [30,38,39]. Although the absolute gains in knowledge were small in this study, over and above the limited sample size, this outcome may also reflect the limitations of a brief intervention delivered in a highly stressful NICU context. Learning under stress can be particularly challenging, as stress impairs memory and cognitive flexibility and may contribute to self-doubt or guilt [40]. The modest improvements observed are consistent with this dynamic and underscore the importance of considering emotional readiness when delivering NICU-based education to parents.

Most births in this study were emergency caesarean sections, followed by vaginal and planned caesarean deliveries. Emergency births, often associated with maternal complications such as hypertension, may heighten maternal stress and reduce receptiveness to education [41,42]. Although birth trauma was not formally assessed, it is likely a relevant factor influencing maternal emotional states and learning capacity [43,44].

Additional contributing factors to stress likely include infant instability, information overload, limited support at home and financial constraints [4,45]. High PSS:NICU scores have been associated with very preterm birth, twin pregnancies, and older maternal age [46], which aligns with our sample: infants had a mean gestational age below 31 weeks and an average birthweight below 1400 g, although most were singletons, mothers averaged 30 years of age.

Several mothers reported limited access to clinical information during the pre-test phase, highlighting communication gaps within the NICU. Addressing these systemic challenges may be as critical as educational interventions for supporting parental confidence and mitigating stress.

### 4.1. Limitations, Challenges, and Considerations for Future Research

This pilot study served as a critical foundation for a larger educational programme. Its primary aim was to design a culturally sensitive, feasible, and acceptable intervention while exploring the contextual and practical considerations of its implementation in our setting. In addition to using validated tools to assess maternal knowledge and stress, the pilot phase provided valuable insights into the contextual suitability of these instruments. Publishing these findings is important, as they inform refinements to both the content and delivery of the subsequent full-scale programme and highlight key considerations for researchers conducting similar work in comparable contexts.

As a pilot study, our sample was not powered to assess whether the outcomes were influenced by gestational age, infant health, maternal characteristics, or education level. Future research could stratify outcomes by gestational age or birth weight, exploring whether these factors influence parental knowledge uptake and stress. Although the majority of participants identified as Black African, they represented diverse ethno-cultural backgrounds, predominantly Setswana. This may limit the generalisability of findings for a broader regional population; particularly where cultural practices may shape parental perceptions and needs in the NICU.

The participants’ education levels ranged from secondary school to post-secondary education, with only four participants holding a degree. This supports tailoring educational content to secondary-level literacy for future interventions.

Some data were missing due to incomplete maternal recall, particularly for variables such as Apgar scores and head circumference measurements, where up to 25 values were missing in the combined samples. Access to infant medical records in future studies would help strengthen data completeness and accuracy.

Although length of stay and severity of illness were not formally measured in this study, anecdotal observations from the research sites suggested that differences in gestational age and birthweight may have influenced maternal engagement and follow-up. Infants with higher gestational age and birthweight appeared to be more medically stable and were often discharged earlier, which may have limited opportunities for mothers to participate fully in the intervention or complete post-discharge measures. In contrast, mothers of smaller or more premature infants tended to experience longer or more intensive hospital stays, during which increased stress and competing caregiving demands may have reduced their ability to engage consistently. These contextual factors could partly explain the variability in engagement and follow-up observed across groups.

Some anecdotal data were collected during the course of the study and may inform the planning of future studies. Recruitment and retention barriers were evident in our study. Although pre-test participation was high, post-test follow-up was hampered by early discharge, distance from the hospital, and financial or logistical constraints. Recruitment was also affected by social dynamics; on some days, a “leader mom” discouraged others from enrolling, underscoring the importance of individual-level engagement strategies in future trials. This situation was addressed through respectful, individualised engagement with the mothers once they were identified. The research team took time to listen to their concerns and explain the study’s purpose, potential benefits, and implications for mothers in similar circumstances. In some cases, this approach helped build trust and encouraged the “leader mom” to participate, which in turn positively influenced others. In other instances, however, the group chose not to participate, and recruitment efforts proceeded with other interested participants.

Mothers faced systemic barriers, such as transportation issues and limited capacity to remain in the hospital after delivery. Mistrust in healthcare systems and the varying levels of health literacy also presented challenges. Building culturally appropriate and trust-based educational relationships is crucial for future success.

Although care was taken to simplify the language, many mothers struggled to articulate their observations and required assistance in doing so. The KPIB tool was time-consuming, sometimes leading to participant fatigue or dropout. Simplified or visual-based tools could enhance comprehension and completion. Incorporating infant clinical data directly from patient records would improve accuracy and reduce reliance on maternal recall. Both the KPIB and PSS: NICU tools were only available in English; however, translation services were available to mothers, which were only used for translation to Setswana.

### 4.2. Implications for Practice and Future Research

To refine and scale parenting educational interventions in the NICU, this pilot study highlights several key areas for development based on observed operational challenges and participant responses.

First, it is critical to stratify educational content according to gestational age, maternal experience, and literacy levels. In our study, a standardised intervention was applied across all mothers; however, the observed feedback and engagement levels suggested that first-time mothers, those with extremely preterm infants, and mothers with lower literacy levels engaged differently with the material. Tailoring content would allow for more targeted and meaningful learning, potentially improving both knowledge acquisition and confidence.

Second, the study was conducted in two hospitals serving semi-urban populations with some demographic and cultural homogeneity. For broader applicability, future iterations should include more diverse cultural and socioeconomic groups. This is especially relevant in South Africa’s public healthcare sector, where linguistic, cultural, and resource disparities can influence how health information is received and acted upon by the public.

The importance of tracking how parental knowledge and stress evolve over time became apparent during follow-up attempts post-discharge. Although some knowledge gains were observed immediately after the intervention, the sustainability of these gains and their long-term impact on maternal stress remain unclear. Ongoing measurement beyond the NICU stay would provide a clearer picture of the intervention’s effectiveness over time and support continuity of care into the home setting.

Given that the severity of the infant’s illness and the length of hospital stay appeared to influence the level of attention and emotional capacity mothers devoted to the intervention, future research should examine these factors as potential moderators of intervention effectiveness to support more personalised delivery and timing.

Our experience with the current assessment tools also points to the need for refinement. Some mothers found the knowledge questionnaire dense or abstract, especially those with lower health literacy or less fluency in English. Furthermore, given the high and increasing maternal stress levels observed, future iterations of the intervention could expand components that support stress management and provide greater psychosocial support. More visually engaging, simplified, and culturally contextualised tools may improve the quality of data collected in the future. Additionally, the questionnaire could become a tool to help mothers understand the behaviours of their infants, rather than information that contributes to anxiety.

Lastly, the value of real-time embedded support became clear, especially in the intervention arm where structured sessions were delivered. Mothers frequently expressed appreciation for the relational and practical support they received during these sessions, an element that was absent from the booklet-only arm. Embedding such support into routine care, particularly in resource-constrained environments, could increase accessibility and the sustained impact of the intervention without requiring large-scale resource additions.

Together, these insights underline the importance of responsive, inclusive, and contextually grounded strategies for delivering parenting education in the NICU, especially within the constraints and realities of public-sector healthcare in middle-income countries.

## 5. Conclusions

Our findings suggest that structured educational interventions, particularly those combined with facilitated sessions, have the potential to enhance maternal knowledge during the critical NICU period. Although parental stress increased across all groups over time, the facilitated intervention was associated with a smaller increase in stress, indicating its potential to support early parenting, promote infant development, and mitigate long-term parental anxiety. This study provides foundational insights into parenting education in a public South African NICU and emphasises the need for accessible, culturally relevant, and well-supported interventions. Future work should focus on scaling this approach, optimising delivery, and evaluating long-term outcomes.

## Figures and Tables

**Table 1:** Maternal characteristics by hospital.

Variable	Hospital A (*n* = 30)	Hospital B (*n* = 30)	Total (*N* = 60)
Maternal age (years), mean ± SD	30.3 ± 7.0	29.7 ± 7.0	30.0 ± 7.0
Marital status, *n* (%)			
Single	29 (96.7%)	24 (80.0%)	53 (88.3%)
Married	1 (3.3%)	5 (16.7%)	6 (10.0%)
Separated	0 (0%)	1 (3.3%)	1 (1.7%)
Number of pregnancies, mean ± SD	3.0 ± 1.7	2.4 ± 1.5	2.7 ± 1.6
Ethnicity, *n* (%)			
African	30 (100%)	28 (93.3%)	58 (96.7%)
Coloured	0 (0%)	1 (3.3%)	1 (1.7%)
White	0 (0%)	1 (3.3%)	1 (1.7%)
Pregnancy complications, *n* (%)			
None reported	19 (63.3%)	13 (43.3%)	32 (53.3%)
High blood pressure	5 (16.7%)	7 (23.3%)	12 (20.0%)
Foetal distress	3 (10.0%)	4 (13.3%)	7 (11.7%)
Bleeding	2 (6.7%)	0 (0%)	2 (3.3%)
Other	0 (0%)	5 (16.7%)	5 (8.3%)
Not reported	1 (3.3%)	1 (3.3%)	2 (3.3%)
Mode of delivery, *n* (%)			
Vaginal	15 (50.0%)	10 (33.3%)	25 (41.7%)
Planned caesarean section	3 (10.0%)	3 (10.0%)	6 (10.0%)
Emergency caesarean section	12 (40.0%)	17 (56.7%)	29 (48.3%)
Type of birth, *n* (%)			
Singleton	25 (83.3%)	25 (83.3%)	50 (83.3%)
Twins	5 (16.7%)	5 (16.7%)	10 (16.7%)

Note. Values presented as mean ± standard deviation (SD) for continuous variables and frequency (percentage) for categorical variables.

**Table 2:** Maternal characteristics by study arm.

Variable	Arm 1 (*n* = 20)	Arm 2 (*n* = 20)	Arm 3 (*n* = 20)
Maternal age (years), mean ± SD	30.2 ± 7.6	31.2 ± 6.1	27.9 ± 7.2
Marital status, *n* (%)			
Single	17 (85.0%)	17 (85.0%)	19 (95.0%)
Married	2 (10.0%)	3 (15.0%)	1 (5.0%)
Separated	1 (5.0%)	0 (0%)	0 (0%)
Number of pregnancies, mean ± SD	2.6 ± 1.5	2.7 ± 1.8	3.0 ± 1.4
Ethnicity, *n* (%)			
African	19 (95.0%)	19 (95.0%)	20 (100%)
Coloured	0 (0%)	1 (5.0%)	0 (0%)
White	1 (5.0%)	0 (0%)	0 (0%)
Pregnancy complications, *n* (%)			
None reported	10 (50.0%)	9 (45.0%)	13 (65.0%)
High blood pressure	5 (25.0%)	6 (30.0%)	1 (5.0%)
Foetal distress	2 (10.0%)	2 (10.0%)	3 (15.0%)
Bleeding	2 (10.0%)	0 (0%)	0 (0%)
Other	0 (0%)	3 (15.0%)	2 (10.0%)
Not reported	1 (5.0%)	0 (0%)	1 (5.0%)
Mode of delivery, *n* (%)			
Vaginal	10 (50.0%)	7 (35.0%)	8 (40.0%)
Planned caesarean section	2 (10.0%)	2 (10.0%)	2 (10.0%)
Emergency caesarean section	8 (40.0%)	11 (55.0%)	10 (50.0%)
Type of birth, *n* (%)			
Singleton	17 (85.0%)	16 (80.0%)	17 (85.0%)
Twins	3 (15.0%)	4 (20.0%)	3 (15.0%)

Note. Values presented as mean ± standard deviation (SD) for continuous variables and frequency (percentage) for categorical variables.

**Table 3:** Infant characteristics by hospital.

Variable	Hospital A (*n* = 34)	Hospital B (*n* = 34)	Total * (*N* = 68)
Gestational age (weeks), mean ± SD	30.1 ± 2.7 (*n* = 34)	31.1 ± 2.6 (*n* = 34)	30.6 ± 2.7 (*N* = 68)
Birth weight (g), mean ± SD	1402 ± 380 (*n* = 34)	1300 ± 428 (*n* = 34)	1351 ± 405 (*N* = 68)
Head circumference (cm), mean ± SD	27.9 ± 3.0 (*n* = 26)	28.6 ± 3.3 (*n* = 7)	28.1 ± 3.1 (*N*= 33)
Apgar score at 1 min, mean ± SD	6.9 ± 2.1 (*n* = 25)	7.4 ± 2.6 (*n* = 31)	7.2 ± 2.4 (*N* = 56)
Apgar score at 5 min, mean ± SD	8.5 ± 1.1 (*n* = 24)	8.3 ± 2.3 (*n* = 31)	8.4 ± 1.9 (*N* = 55)
Sex, *n* (%)			
Male	14 (41.2%)	16 (47.1%)	30 (44.1%)
Female	20 (58.8%)	18 (52.9%)	38 (55.9%)

Note. Values presented as mean ± standard deviation (SD) for continuous variables and frequency (percentage) for categorical variables. * Total column values are pooled using available data for each variable; therefore, *n* varies per row.

**Table 4:** Infant characteristics by study arm.

Variable	Arm 1 (*n* = 22)	Arm 2 (*n* = 23)	Arm 3 (*n* = 23)
Gestational age (weeks), mean ± SD	29.8 ± 2.6 (*n* = 22)	31.3 ± 2.4 (*n* = 23)	30.7 ± 2.8 (*n* = 23)
Birth weight (g), mean ± SD	1249 ± 269 (*n* = 22)	1424 ± 401 (*n* = 23)	1361 ± 498 (*n* = 23)
Head circumference (cm), mean ± SD	27.4 ± 2.5 (*n* = 18)	30.1 ± 3.6 (*n* = 13)	27.9 ± 3.4 (*n* = 12)
Apgar score at 1 min, mean ± SD	7.0 ± 2.5 (*n* = 19)	7.1 ± 2.1 (*n* = 20)	7.3 ± 2.5 (*n* = 17)
Apgar score at 5 min, mean ± SD	8.4 ± 1.7 (*n* = 19)	8.4 ± 2.0 (*n* = 20)	8.3 ± 2.3 (*n* = 16)
Sex, *n* (%)			
Male	8 (36.4%)	11 (47.8%)	11 (47.8%)
Female	14 (63.6%)	12 (52.2%)	12 (52.2%)

Note. Values presented as mean ± standard deviation (SD) for continuous variables and frequency (percentage) for categorical variables.

**Table 5:** KPIB pre- and post-test scores.

	Hospital A				Hospital B				Hospital A and B (Pooled Data)			
	Arm 1	Arm 2	Arm 3	Total	Arm 1	Arm 2	Arm 3	Total	Arm 1	Arm 2	Arm 3	Total
Pre-test KPIB												
Average Score out of 36	11.7	11.8	9.9	11.1	12.5	12.5	12.1	12.4	12.1	12.2	11	11.7
Average percentage	33%	33%	28%	31%	35%	35%	34%	34%	33.6%	33.5%	30.6%	32.6%
Post-test KPIB												
Average Score out of 36	11.2	12.7	11.6	11.8	13.4	12.5	13.3	12.93	11.8	12.7	12.5	12.4
Average percentage	31%	35%	32%	33%	37%	35%	37%	36%	32.9%	35.4%	34.6%	34.3%
Change between pre- and post-test (in %)	−2%	+2%	+4%	+2%	+2%	0%	+3%	+2%	−0.7%	+1.9%	+4.0%	+1.7%

**Table 6:** KPIB ANOVA.

Effect	df1	df2	F	p	ηp^2^
Time	1	57	1.88	0.176	0.03
Group	2	57	0.19	0.828	0.01
Time × Group	2	57	0.78	0.462	0.03

**Table 7:** NICU:PSS pre- and post-test scores.

	Hospital A				Hospital B				Hospital A and B (Pooled Data)			
	Arm 1	Arm 2	Arm 3	Total	Arm 1	Arm 2	Arm 3	Total	Arm 1	Arm 2	Arm 3	Total
Pre-test NICU:PSS												
Average Score out of 170	128.7	112.6	99.1	113.5	88.3	101.9	104.2	98.13	108.5	107.3	101.5	105.1
Average percentage	76%	66%	58%	67%	52%	60%	61%	58%	63.8%	63.1%	59.8%	62.4%
Post-test NICU:PSS												
Average Score out of 170	119.9	121.5	105.3	115.6	113.6	130	110.6	118.07	116.8	125.8	108.0	116.8
Average percentage	71%	71%	62%	68%	67%	76%	65%	69%	68.7%	74.0%	63.5%	68.7%
Change between pre- and post-test	−4%	+5%	+4%	+1%	+15%	+16%	+4%	+11%	+4.9%	+10.9%	+3.7%	+6.3%

**Table 8:** NICU:PSS ANOVA.

Effect	df1	df2	F	p	ηp^2^
Time	1	57	8.40	0.005	0.13
Group	2	57	0.75	0.479	0.03
Time × Group	2	57	0.99	0.378	0.03

## Data Availability

The intervention materials (e.g., programme content, handouts, facilitator guidelines) are not publicly available due to copyright and ongoing development. However, they can be made available from the corresponding author upon reasonable request for academic or implementation purposes.
